# Dietary supplements could prevent cardiometabolic syndrome: Are they safe and reliable enough for disease prevention and health promotion?

**DOI:** 10.3389/fcvm.2023.1091327

**Published:** 2023-03-22

**Authors:** Istvan G. Télessy, Harpal S. Buttar, Douglas W. Wilson, Charles Odilichukwu R. Okpala

**Affiliations:** ^1^ Faculty of Pharmacy, Department of Pharmaceutics, University of Pécs, Pécs, Hungary; ^2^ Department of Pathology & Laboratory Medicine, School of Medicine, University of Ottawa, Ottawa, Canada; ^3^ Formerly, School of Medicine Pharmacy and Health, Durham University, Durham, UK; ^4^ Centre for Ageing and Dementia Research, Swansea University, Swansea, United Kingdom; ^5^ Faculty of Biotechnology and Food Sciences, Wrocław University of Environmental and Life Sciences, Wrocław, Poland; ^6^ UGA Cooperative Extension, College of Agricultural and Environmental Sciences, University of Georgia Athens, Athens, GA, United States

**Keywords:** food supplements, fish oil, coenzyme-Q10, magnesium, indication, formulation, dosage

## Abstract

Dietary supplements (DS) and their purchase is often based on a consumer's personal choice and advertisements. The associated DS regulations, particularly in manufacturing and marketing, are far more flexible and permissive than that of the well-regulated prescription pharmaceuticals. However, the adverse health effects associated with the inadvertent use of mega-doses of DS are not well understood. The demand for DS, nutraceuticals, and herbal remedies has experienced an upswing during the past two to three decades, and global product sales have thrived. More so, the prevention of cardiometabolic syndrome (CMS) and related disorders like diabetes mellitus, obesity, hypertension, and serum lipid abnormalities, as well as of other noncommunicable diseases (NCDs), is of highest health care priority globally, since these disorders impose very high economic burdens on health care systems and society. In this review, we argue why DS could prevent cardiometabolic syndrome, by providing the potential benefits and risks associated with them, especially self-medication considering their intake by the public at large. Good manufacturing practices and quality control are absolutely necessary for the manufacture of DS products, and proper labeling is needed regarding the optimal dose schedules of various DS and bioactive ingredients. Specific examples are used to underscore the indications and dosage recommendations made for the marketing and promotion of fish oil, coenzyme Q10, and Mg-containing products for the prevention of cardiometabolic syndrome.

## Introduction

Cardiometabolic syndrome (CMS) represents a cluster of events associated with distortion of carbohydrate and fat metabolism as well as macro- and micro-vascular complications. Basically, CMS is characterized by hypertension, hyperlipidemia, atherosclerosis, increased risk of coronary heart disease and stroke, hyperglycemia, insulin resistance, and some cancers. In the majority of cases, an increase of abdominal fat, diabetes mellitus, and unhealthy dietary habits promote this syndrome. The leading diagnostic signal is abdominal obesity, which serves as a warning of an elevated risk of CMS and the need to search for any hidden CMS parameters. Dietary supplements (DS), which include food supplements and nutraceuticals have gained much popularity for the management of weight and the detrimental sequelae associated with obesity and diabetes. More so, DS are widely used all over the world by teenagers and adults, especially by elderly individuals and particular groups of people such as athletes and body-builders. In 2021, global DS sales were estimated to be 151.9 billion USD (1). The demand for DS is rapidly growing all over the world, because people think DS consumption could prevent non-communicable diseases (NCD), such as obesity, diabetes, and cardiovascular and neurological disorders, as well as help to maintain good health and wellbeing (2). In contrast, Chen and colleagues amongst others have mentioned that the use of DS was neither associated with any health benefits nor a reduction of mortality (3, 4). In this review, we are attempting to answer a number of questions, namely: what is hidden beneath the controversy surrounding DS? Perhaps it might be the uncontrolled availability of DS, or the media hype created through advertisements, or the time-consuming visits by consumers to their doctor's office. Or perhaps it is due to the missing information for the consumer regarding the safety of DS, or inappropriate self-medication with natural health products. It has been reported that less than a quarter of DS were recommended by doctors or other healthcare professionals, and more than 75% consumers bought DS of their own choice (5). These observations raise important questions: do DS provide real health benefits? Is the labeled-information reliable? Are they safe enough when used for long-term periods? More so, the adverse health effects associated with the inadvertent use of mega-doses of DS are not well understood by the lay public as well as by so-called sophisticated buyers. Nowadays, botanical products containing a broad spectrum of compounds are used by consumers due to their natural origin and availability. Many people buy DS from supermarkets instead of registered food and drug stores where trained health professionals can answer their questions and explain the risks and benefits associated with the DS. Additionally, the excessive intake of exogenous antioxidants and anti-inflammatory products can cause health hazards because higher dosages of antioxidant supplements can, for example, suppress or prevent some physiological functions of free radicals that are needed for cell signaling in tissues and organs (e.g., in the skeletal muscles, heart, gut, liver, and central nervous system) (6–8). On the one hand, substantial information is available about the adulteration of DS with synthetic drugs and heavy metals (As, Pb, Hg, Cd), sub-standard and counterfeit products, and the issues/concerns about the risks of internet-commerce is well-founded (9–11). On the other hand, the advertising media (e.g., TV, newspapers, home magazines, and the internet) encourages people to buy more and more DS and nutraceuticals for self-medication and health promotion (12, 13). There are probable reasons linked to peoples' choice of buying DS and point out the weaknesses of decision-making. We provide here three examples of commonly used DS (e.g., fish oil, coenzyme Q10, and Mg-containing products) and their bioactive dietary ingredients. Quite often, these DS are consumed for the prevention of CMS. In this review, we debate why DS could prevent or slow down progress of cardiometabolic syndrome by providing the potential benefits and risks associated with it, especially regarding self-medication considering their intake by the public at large. We have selected one representative of each of the major preventive groups, that is, anti-inflammatory, antioxidant, and microelements, used to diminish the risk of developing CMS. Our main reasons for selecting these examples are as follows. Firstly, fish oil is known as anti-inflammatory foodstuff and nutraceutical (14–16). Secondly, coenzyme Q10 has been recommended as an effective remedy for the management of various cardiovascular diseases, primarily due to its antioxidant and anti-inflammation effects (17–19); it also plays an important preventive role in the occurrence and pathogenesis of diabetes mellitus (T2DM) (20). Finally, several published reports have shown that magnesium-containing products are useful in the prevention and treatment of CMS, predominantly based on its physiological role in the functioning of metalloproteins in the body (21–24).

### Motivations for self-medication with DS

The earliest signs of increased risk of cardiometabolic syndrome are overweight or obesity. This means 48.7% and 68.3% of overweight (BMI > 25 to  < 30) and obese (BMI > 30) subjects, respectively, are metabolically abnormal (25). If obesity occurs in childhood or adolescence, there is a high likelihood of its persistence during adulthood, and this can be considered as an obvious driver for counteraction. However, according to the recent study by Zavala et al., 80% of 6,400 participants reported at least one barrier to healthy eating and 78% at least one barrier to performing physical activity (26). Even in developed countries, there are barriers associated with the affordability of a healthy diet (27). Changes in lifestyle and low-calorie diets as well as intensive leisure-time physical activity seem not to be first choice interventions against weight gain and CMS, and, therefore, the majority of people look for simpler resolutions, e.g., buy and consume DS or nutraceuticals. Moreover, the primary sources of information would continue to remain the internet, family, and friends for the majority of people, bearing in mind that not all such information would always be reliable. As advertisements push DS into the spotlight and they are available without restraint in various shops, many people turn to these convenient solutions. Also, comfort has a powerful grip, even if it is acknowledged that the scientific-based planning of interventions may offer more success (28–30). However, the high rate of DS consumption may also stem from the conviction that a preparation which looks like a drug must have therapeutic properties as well (31). Furthermore, medical or surgical interventions are usually recommended just in cases of obesity with concurrent chronic disease or severe obesity (32). Whereas, older adults are more likely than younger individuals to report site-specific motivations like the heart, bones and joints, and eye health (4, 33). It is, however, clear that early diagnosis and indication is important to employ lifestyle and risk factor modification, the latter, for example, with the help of dietary supplements.

### Rationale for dietary supplement intake

The primary question arises regarding taking DS: for what purpose should one take DS? For instance, the good health promotion effects of DS can be expected if they are used according to recommendations. In case of DS, a wide variety of medical indications must not be declared on the package labels. However, many ingredients containing DS list the main active pharmaceutical component as well and therefore the indication is indirectly validated. Pharmaceuticals often serve as beacons of light for DS users. Wrongly interpreted indications can counteract the health benefit expectations. The main ingredients of fish oil (EPA and DHA) also exist as ingredients of approved pharmaceuticals, indicating fish oil is a reliable tool for the prevention and management of CMS (34, 35). But none of the EPA-DHA-containing pharmaceutical-product information have weight management among the indications, thus fish oil supplementation is not suitable for weight loss or as a slimming diet (36). However, fish oil DS products are useful in the prevention of the cardiovascular components of cardiometabolic syndrome. A dose-response relationship was shown between an increasing level of n-3 PUFA biomarkers and lower risk of cardiovascular diseases (16, 37).

The predominantly non-pharmaceutical DS containing coenzyme Q10 on the market are promoted as if prospective randomized controlled multicenter clinical trials are a trustworthy indication of its effects (38, 39). The FDA did not approve it to treat any medical condition, yet for the prevention of the cardiovascular components of metabolic syndrome, good quality studies support its beneficial effects (18, 40, 41). With regard to other individual CMS-components, coenzyme Q10 seems to be useful in the co-treatment of glycemic control (prediabetes and diabetes patients) as well (42). Of note, coenzyme Q10 supplementation has a beneficial effect on glycemic control of Type 2 diabetes mellitus but not Type 1 diabetes mellitus (43). As a matter of fact, use of coenzyme Q10 is reasonable in all cases of Q10 deficiency. This compound is synthesized in the body; however, endogenous biosynthesis tends to decline with age and tissue concentration may be compromised in many pathological states, e.g., diabetes, dyslipidemia, and CVD (44, 45). A lot of consumers take magnesium supplements for relieving leg cramps. To date, no evidence exists for its effectiveness in this indication, yet this misconception often overwrites normal use of magnesium-containing pharmaceuticals (46). Metabolic syndrome is, however, a real indication of magnesium administration (22). Potentially, hypo- and hypermagnesemia should be considered as dangerous, and, under special situations, both extreme deviations may worsen the outcomes of hospital mortality in a dose-dependent manner (47).

### What about the bioactive ingredients?

In many places around the globe, the assortment of ingredients (the composition) of DS during the development process are not restricted; however, there are some national restrictions. The United States FDA and EU EMA regulate ingredients which are prohibited in DS; however, product samples are not submitted to any safety assessment prior to commercialization. Therefore, qualitative and quantitative errors and non-conformities emerge only after public announcements regarding quality defects or other problems (48, 49). Forbidden chemicals occur in DS due to adulteration or contamination (50–52). Unfortunately, there is a growing trend of the falsification of DS, especially with the thriving of online commerce (53, 54). In these cases, there is a difference between what is on the label and in the bottle; thus, the fake DSs are often of poor quality and sometimes a threat to public health. The adulterations are usually made using active pharmaceutical ingredients, but in phytotherapeutic products this usually occurs with other plant-origin components as well (55–58). In 1994–95, polychlorinated biphenyls were found to have contaminated 38 fish oil DS originating from 15 different countries (59). Apart from adulterations and contaminations, other problems may also cause confusion in customers (60). Package information and labels are often short and poorly written, and open to misapprehension (61).

The chemical representation is a key factor in this respect, e.g., in Q10 products, ubiquinone, the oxidized form of Q10, is a more common ingredient in commercial supplements; however, reduced ubiquinol (the physiologically active form in the Q10 redox system) appears to be a better form to promote the patients' Q10 status (62). The salt forms of magnesium-containing dietary supplements pose a similar problem, as inorganic formulations are less bioavailable than organic ones (63). But even within inorganic forms, there are differences: the oxide form (MgO) has lower bioavailability while the others (sulfate, chloride) have acceptability. Also, in case of fish oil, there are significant differences in the formulation composition. Most often, high levels of saturated fatty acids have been detected in fish oil DS (64). The n-3 polyunsaturated fatty acids (n3-PUFAs), such as eicosapentaenoic acid (EPA) and docosahexaenoic acid (DHA), are the important components of fish oil but n3-PUFAs are susceptible to oxidation and under sunlight or UV radiation can lose their effectiveness. A study from New Zealand reported that more than 80% of tested fish oil products were found to contain high amounts of peroxide-levels, that is, above the industry standards, thereby indicating the poor quality of the fish oil (65). The storage conditions and shelf-life of fish oil are important considerations for the consumer's product selection, but what happens during manufacturing and in the supply-chain before purchase is hidden from consumers (66).

### Quantity declaration as a trap?

In the European Union, nutrition declaration for vitamins and minerals is regulated by regulations 1924/2006/EC and 1925/2006/EC (67). According to these regulations, the accepted tolerance for differences between the nutrient values declared on the label and those established in the course of official laboratory control is +50% and −20% for minerals in food supplements. This is a substantive deviation from pharmaceuticals. Most shoppers are not aware that in case of dietary supplements, the regulations are much less strict than those in pharmaceuticals. Therefore, if customers believed 100 mg was really 100 mg, this can lead to misapprehension. For example, in case of magnesium-containing products, this can make 250 mg of “declared content” under correct manufacturing conditions contain limits from 200 mg to 375 mg. This does not allude to poor quality but the range allowed by law.

Research has shown that in a part of the population, serum magnesium-level is too low, lower than 1.4 mg/dl (ca. 0.65 mmol/L), the cut-off for normal value. Hypomagnesemia develops secondary to decreased intake (e.g., starvation, alcohol consumption) or following medications (e.g., diuretics or PPI aminoglycosides). 2% of the general population, 30%–80% of alcohol-use disorders, and 25% of diabetes patients are prone to hypomagnesemia. The dietary magnesium intake is negatively correlated with development of CMS (68). For asymptomatic patients, those who have only night leg cramps, or those at risk of CMS—and the vast majority of individuals taking magnesium in the form of dietary supplements belong to these groups—the recommended daily dose is 300–500 mg, up to a maximum of 4 g/day if kidney function is not compromised (22). In our opinion, prolonged or permanent Mg-restriction as well as above mentioned quantitative deviations may cause problems because the expected ingestion of magnesium will not be fulfilled. The salt form also may be misleading: MgO contains ca. 60% of elementary Mg but magnesium citrate contains 16% magnesium and just 5.4% Mg-gluconate. Therefore, pure magnesium-content should be taken into account and not the amount of the salt-form.

In case of fish oil, sometimes only n-3 PUFA content is emphasized for health promotion on labels or in advertisements. However, alpha-linolenic acid (ALA) is basically different from eicosapentaenic acid (EPA) and docosahexaenic acid (DHA). ALA is mainly from plant origin, whereas EPA and DHA are from fish-origin n-3-PUFAs (69). ALA is theoretically a precursor of EPA but in the human organism just a small proportion of ALA is converted to EPA, and the rest is stored or burnt for energy production like any other fatty acid (70). Finally, ALA is not as highly valued as EPA and DHA from a cardioprotective point of view.

### Dietary supplement dosage and formulation impact

Among the key basic questions of medical therapy is “dosage”. For food supplements, there appears to be no pharmacological dosage recommendation. Despite this, national authorities continue to provide the recommended daily allowance (RDA). Even on labels, many manufacturers provide recommendations for daily dose. It should be noted that the RDA data are usually age- and condition-dependent. And in order to minimize customers expenditure and gain an advantage over rivals, the manufacturers often indicate lower daily dosages on the label. For example, for fish oil, the pharmacologically recommended therapeutic dosage for hypertriglyceridemia, part of CMS, is 2 g per day or more with a minimum-content of 1,600 mg of eicosapentaenic + docosahexaenic acids. In contrast, many commercial fish oil capsules contain less than 500 mg of this combination and the recommended dose is 1–2 capsules daily. A study in 2018 explored that, in New Zealand, 74% of fish-oil consumers do not take the minimally recommended 400–600 mg EPA + DHA or more per day (71). Obviously, this amount cannot ensure the expected benefit. Therefore, consultation with an MD, dietician, or pharmacist is advisable. For prevention, slightly lower doses of the abovementioned therapeutic dose can be taken; however, this is effective only in long-term use, i.e., years in the case of fish oil, because the anti-inflammatory effect due to the modification of the ratio of pro- and anti-inflammatory cytokine production takes time (72).

The therapeutic dose of coenzyme Q10 is recommended to be between 100 and 200 mg/day. This substance is very safe as it is an endogenous molecule, therefore, it is quite difficult to overdose. In case of low coenzyme Q10 levels, for prevention, lower doses (100 mg/day) are also adequate. For comparison: from a healthy diet, only 2–10 mg/day can be obtained, which is enough only in the presence of redox balance (73). Magnesium is a special case, because its absorption decreases as the dose increases (74). Mg absorption is better when acidic salt is the source (Mg-acetate, -aspartate, Mg-citrate, Mg-orotate) and when the mineral is taken in low doses throughout the day in comparison to a single high dose per day. That is why a preference for retard-formulations is recommended (75). The recommended daily dose for prevention is ca. 300–500 mg.

Most consumers only look for the bioactive ingredients and the amounts of bioactive substances. As the LADME (liberation-absorption-distribution-metabolism-excretion) process is similar for pharmaceuticals and dietary supplements, the formulation that determines the liberation of the main ingredient from the dosage form (tablet-capsule-suspension-etc.) plays a critical role from a utility point of view. The bioavailability, i.e., the proportion of active substance reaching the systemic circulation is crucial from an effectiveness point of view. As a DS is not usually subject to a bioavailability test, relevant differences may arise in this respect as well. Fine et al. observed that the enteric coating of the magnesium tablet impairs the bioavailability of magnesium (74). Inadvertently, most high-dose Mg-preparations release their content in 1–2 h, but the absorption process takes time, thus leaving a larger fraction of Mg unabsorbed in the gut resulting in osmotic diarrhea. In contrast, specific formulations may prolong the release to 6 h or more, and thereby increase the absorption to near 100% (76).

In case of Q10, there is a significant difference among the various formulations (77, 78) as well. In animal pharmacokinetic studies, three different formulations showed a three to six-fold AUC-increase in bioavailability of Q10 with the same active constituent (79). It was reported that coenzyme Q10 in an oleogel formula is more stable than other gels with lecithin surfactant (80). Soy protein-encapsulated fish oil masks fish oil flavors and protects it from lipid oxidation in contrast to traditional capsulated formulas (81). Carboxylic acid formulation containing EPA + DHA could not reach the cardiovascular effectiveness of other EPA + DHA products in clinical trials (82). The above examples demonstrated that it is not only the amount of active substance that plays a role in the value of a dietary supplement; facilities, human resources, know-how, and technical preparedness are decisive for a manufacturer. Moreover, their production strategy (e.g., to strive for high quality management or choose less expensive processing and sub-quality raw materials) determines the quality.

## Concluding remarks: “Innocent until proven guilty”

DS manufacturers tend to produce and market safe and reliable products, as it is their clear interest and intention to do so in the long-term. Nowadays, however, there are inconformity issues with DSs. Microbial contamination, heavy metal contamination, prescription drug adulteration, substitution of active plant varieties, or fraudulently underdelivering ingredients are among the major challenges (83–85), some of which have emerged due to improper design or production control as well as possibly “calculated errors”. It is imperative that these negative affairs should be evaluated, considering that one situation would likely differ from another. Primarily, in the opinion of the authors herein, DS should remain as DS and similar properties and reliability to those of pharmaceuticals should not be expected. Furthermore, DS should not be recommended as an alternative to drugs where guidelines recommend treatment. More so, the results of dietary supplement products cannot be transferred to others with the same or similar active substance. Clinical trials, multicentric studies, and meta-analyses demonstrate controversial results in CMS prevention with identical active ingredients but hardly comparable products. Notably, there is need to consider that, prior to the purchase of DS, pharmacists or dietitians ought to be consulted in order to ensure appropriate knowledge about the given/specific product is properly acquired by the client/customer/consumer. In the case of CMS however, dietary supplements could generally provide a moderate preventive value, as does healthy eating and lifestyle. They are neither toxic nor induce more complications compared to placebo controls and, therefore, can be purchased without any prescription. Moreover, from a cost-effectiveness point of view, they are accepted by the public and medical professionals (86, 87).
